# Transverse Dental Arch Dimensions and Body Mass Index in Pediatric Patients with Marfan Syndrome: A Case–Control Observational Study

**DOI:** 10.3390/children13081112

**Published:** 2026-08-20

**Authors:** Arianna Malara, Maria Driade Anastasio, Fabio Bertoldo, Giuseppina Laganà, Raffaella Docimo

**Affiliations:** 1 Department of Pediatric Dentistry (0–14 Years Old), Policlinico of Rome “Tor Vergata”, 00133 Rome, Italy; driade_93@hotmail.it (M.D.A.); g.lagana@unilink.it (G.L.); raffaella.docimo@ptvonline.it (R.D.); 2 Cardiac Surgery Division, Policlinico of Rome “Tor Vergata”, 00133 Rome, Italy; 3 Department of Life, Health, and Health Professions Sciences, Link Campus University, 00165 Rome, Italy

**Keywords:** Marfan syndrome, body mass index (BMI), maxillary transverse deficiency, growing patients

## Abstract

**Objectives**: To describe BMI-for-age and transverse dental arch measurements in growing children with Marfan syndrome (MFS) and a non-Marfan pediatric comparison group. **Materials and Methods**: A retrospective case–control observational study included 41 children aged 6–11 years: 15 patients with MFS (7 males, 8 females; mean age 8.8 ± 1.5 years) and 26 non-Marfan controls (10 males, 16 females; mean age 8.9 ± 1.6 years). Anthropometric parameters and BMI-for-age z-scores were assessed using the WHO 2007 Growth Reference. Digital dental models were used to measure intermolar width (IMW), intercanine width (ICW), and the IMW/ICW ratio. Descriptive statistics, exploratory between-group comparisons, and Pearson correlation analyses were performed. **Results**: Children with MFS showed significantly lower BMI and BMI-for-age z-scores than controls, although BMI-for-age values generally remained within the normal range. Height values were consistently elevated, reflecting the characteristic growth pattern of MFS. The MFS group showed a significantly smaller mean ICW than controls, whereas the difference in IMW was not statistically significant. The IMW/ICW ratio was also evaluated to characterize transverse dental arch proportions. Pearson correlation analyses showed no significant associations between BMI-for-age z-score and IMW or ICW in either group. In controls, age was positively correlated with BMI-for-age z-score and IMW was positively correlated with ICW; these associations were not observed between BMI-for-age z-score and dental measurements in the MFS group. **Conclusions**: Children with MFS showed a leaner anthropometric profile and a significantly smaller mean ICW, whereas the difference in IMW was not statistically significant. These preliminary findings should be interpreted cautiously given the small sample size and exploratory nature of the study. Further longitudinal studies with larger cohorts are warranted.

## 1. Introduction

Marfan syndrome (MFS) is a rare connective tissue disorder that can involve the heart, blood vessels, lungs, eyes, bones, and ligaments. The condition takes its name from the French pediatrician Antoine Bernard Jean Marfan, who first reported it in 1896 in a 5-year-old girl named Gabrielle. She presented with unusually long, slender limbs—described as “spider-like legs”—a feature known as dolichostenomelia (from the Greek stenos = narrow, slender; melos = limb). Her arms, legs, fingers, and toes were noticeably elongated and thin compared with the rest of her body [1].

MFS is caused by mutations in the FBN1 gene, which encodes the extracellular matrix protein fibrillin-1 and leads to defective microfibrils throughout multiple organ systems [2]. The resultant structural and signaling abnormalities, particularly involving transforming growth factor β, give rise to systemic manifestations that include cardiovascular anomalies, musculoskeletal features, ocular complications, and distinctive craniofacial traits. Genetic defects in FBN1 not only underlie the classical skeletal overgrowth observed in affected individuals but also play a critical role in phenotypic variability and clinical severity [3].

Craniofacial anomalies are frequently observed in patients with MFS and may encompass a combination of dolichocephaly, retrognathia, highly arched and narrow palates, and maxillary constriction, all of which contribute to malocclusion and dental crowding in affected individuals [4]. Quantitative anthropometric analyses have confirmed that specific facial measurements differ significantly between MFS subjects and controls, including variations in facial width and palatal vault morphology [5]. Further three-dimensional evaluations have demonstrated that children and adolescents with MFS frequently exhibit maxillary transverse deficiencies and deeper palatal vaults compared with age- and sex-matched controls [1]. These craniofacial differences are not only of orthodontic importance but also have implications for airway function and occlusal relationships.

Despite extensive characterization of craniofacial features in MFS, less is known about the relationship between systemic growth parameters and craniofacial morphology in affected children. MFS is classically associated with a tall and slender body habitus, but pediatric data on body mass index (BMI) patterns and their potential interaction with craniofacial development remain limited. In addition to the well-recognized craniofacial features, several oral and dental abnormalities have been described in children with Marfan syndrome, including a high-arched palate, dental crowding, enamel defects, root deformities, and altered dental development. These manifestations may interfere with normal occlusal development and orthodontic treatment planning. However, evidence regarding the timing of tooth eruption and premature exfoliation of the primary dentition in pediatric patients with Marfan syndrome remains scarce. Therefore, further investigation of these developmental aspects may improve the understanding of the craniofacial phenotype associated with the syndrome [6].

Growth trajectories in children with MFS may diverge from those observed in typical pediatric populations, raising questions about whether alterations in BMI influence skeletal growth and maxillary morphology [7]. Although high BMI has been linked to changes in craniofacial dimensions in non-syndromic populations, including increased sagittal facial measurements and malocclusion prevalence [8], such associations have not been systematically explored in the context of MFS.

Elucidating the interaction between systemic growth characteristics, such as BMI, and maxillary transverse morphology in pediatric patients with MFS may provide insight into shared developmental pathways linking somatic growth and craniofacial structure, with potential implications for interdisciplinary management and early intervention. Therefore, the present study aimed to describe BMI-for-age and dental-arch transverse measurements in a small retrospective sample of children with Marfan syndrome and a non-Marfan pediatric dental comparison group.

## 2. Materials and Methods

This study was carried out in accordance with the principles set out by the World Medical Association in the 2008 Declaration of Helsinki on medical protocols and ethics, and it was approved by the Ethical Committee of the Policlinico Tor Vergata of Rome (protocol number: 96/26). Written consent was obtained from both parents of every subject included in this study.

For this case–control observational study, data were retrospectively collected from the first examination medical records of patients evaluated at the Department of Pediatric Dentistry, Tor Vergata Hospital, Rome, between January 2025 and January 2026.

The final sample consisted of 41 patients (17 males and 24 females; mean age: 8.9 ± 1.5 years) and was divided into two groups:

• MG (Marfan group): consisted of 15 patients (7 males, 8 females; mean age: 8.8 ± 1.5 years) diagnosed with Marfan syndrome according to established clinical criteria and genetic confirmation. These patients were referred to the Pediatric Dentistry clinical department by the Tor Vergata Hospital Regional Centre for Marfan Syndrome.
• CG (control group): consisted of 26 patients without a diagnosis of Marfan syndrome (10 males, 16 females; mean age: 8.9 ± 1.6 years).

Control subjects were consecutive non-Marfan children evaluated at the Department of Pediatric Dentistry during the same study period and fulfilling the predefined inclusion and exclusion criteria. The higher number of controls was related to the availability of eligible subjects during the study period and was not determined by a predefined matching ratio.

For both groups, the inclusion criteria were age between 6 and 11 years, mixed dentition, and no previous history of orthodontic treatment.

Patients with Marfan syndrome were included, whereas subjects with additional syndromic, genetic, endocrine, metabolic, or chronic systemic conditions that could affect growth, body weight, or dental development, as well as those receiving medications potentially affecting growth or body weight, were excluded. Patients with previous craniofacial interventions or significant craniofacial or dental conditions that could influence craniofacial growth or dental development were also excluded.

### 2.1. Clinical Assessment

All participants underwent a comprehensive dental examination, including: inspection of transverse dental arch characteristics and of clinical palatal morphology, presence of posterior crossbite, and dental arch measurements. For each patient, a digital impression of the dental arches was taken using an iTero intraoral scanner (Align Technology Inc., Tempe, AZ, USA). The .stl files were uploaded into OrthoCAD software (3Shape, Copenhagen, Denmark), version 5.9.0.36, used to measure:

- Intercanine width (ICW): distance between the cusps of the right and left maxillary canines.
- Intermolar width (IMW): distance between the mesiobuccal cusps of the first maxillary molars on each side.

To assess the reliability of the measurements, all digital model measurements were performed by a single operator (M.D.A.). A second examiner (A.M.) independently repeated the measurements. Both examiners were blinded to the participants’ group allocation during the measurement procedure. The entire assessment was repeated after a two-week interval. Systematic error was evaluated by comparing repeated measurements using a paired *t*-test. The magnitude of random error was calculated according to Dahlberg’s formula [8].

### 2.2. Developmental and Medical History Assessment

Information regarding dental eruption and pubertal timing was retrospectively collected through medical history interviews with the parents and review of available clinical records. Premature exfoliation of primary teeth was recorded based on parental report and clinical history, including the affected teeth and the reported age at exfoliation. No specific diagnostic procedures were performed to distinguish premature exfoliation from other possible causes such as trauma, caries, periodontal conditions, or physiological variation; therefore, these data were considered exploratory. Information regarding precocious puberty was also collected through parental medical history reporting. Since standardized endocrinological assessment, Tanner staging, and hormonal evaluation were not available, pubertal timing data were considered retrospective observations and were interpreted with caution.

### 2.3. Anthropometric Evaluation

Height and weight were measured using standardized procedures. Body mass index (BMI) was calculated as weight in kilograms divided by height in meters squared (kg/m^2^). BMI-for-age z-scores were calculated using WHO AnthroPlus software (version 1.0.4; World Health Organization, Geneva, Switzerland) applying the WHO 2007 Growth Reference for children and adolescents aged 5–19 years [9]. Age was entered into the software in years and completed months. The sex-specific WHO 2007 reference population was used for the calculation of BMI-for-age z-scores. In the revised analysis, only BMI-for-age z-scores were reported, and percentile classifications and BMI nutritional categories were removed from the tables to avoid potential inconsistencies between z-scores and percentile-based classifications.

### 2.4. Statistical Analysis

Descriptive statistics were used to summarize demographic, anthropometric, and craniofacial characteristics of the study population. Continuous variables are presented as means and standard deviations (SDs), whereas categorical variables are reported as frequencies and percentages. Given the exploratory nature of the study and the limited sample size of the Marfan syndrome cohort, statistical analyses were interpreted cautiously. Exploratory comparisons between the Marfan syndrome group and the control group were performed for selected continuous variables using independent samples *t*-tests, with mean differences and 95% confidence intervals reported. In addition, exploratory Pearson correlation analyses were conducted to assess potential linear associations between BMI-for-age z-score and maxillary transverse dental arch measurements (IMW and ICW) within each group. These analyses were considered hypothesis-generating and were interpreted in the context of the limited sample size. Statistical analyses were performed using IBM SPSS Statistics for Windows, Version 26 (IBM Corp., Armonk, NY, USA).

### 2.5. Sample Size Consideration

Given the exploratory nature of the correlation analysis and the rarity of Marfan syndrome in pediatric populations, a post hoc sample-size assessment was performed to contextualize the statistical limitations of the study. Assuming a moderate linear association between BMI and maxillary transverse dimensions (r = 0.40), a sample size of approximately 45 subjects per group would be required to achieve 80% power with a two-sided α = 0.05. Therefore, the present findings should be interpreted as preliminary and hypothesis-generating, and further studies with larger cohorts are required to confirm these observations.

## 3. Results

A total of 41 children aged 6–11 years were included, comprising 15 patients with Marfan syndrome (MG) and 26 with no Marfan syndrome (CG). The MG consisted of 7 males and 8 females (mean age 8.8 ± 1.5 years), whereas the CG included 10 males and 16 females (mean age 8.9 ± 1.6 years). All subjects were in the mixed dentition stage and fulfilled the inclusion criteria.

### 3.1. Anthropometric Descriptive Analysis in Marfan Group (MG)

The Marfan syndrome group included 15 children aged 6.1–11.2 years. Mean BMI was 15.03 ± 1.96 kg/m^2^, ranging from 13.22 to 19.37 kg/m^2^. BMI-for-age z-scores ranged from −1.72 to +1.02 (Table 1), with a mean value of −0.83 ± 0.87 (Table 2). Overall, children with Marfan syndrome showed a leaner anthropometric profile than controls, although BMI-for-age values generally remained within the normal range. Height values were consistently elevated, reflecting the characteristic tall stature associated with Marfan syndrome [10,11].

### 3.2. Anthropometric Descriptive Analysis in Control Group (CG)

The control group displayed a broader anthropometric distribution (Table 3). Mean BMI was 20.01 ± 2.36 kg/m^2^, ranging from 14.97 to 23.39 kg/m^2^, while BMI-for-age z-scores ranged from −0.22 to +2.33, with a mean value of 1.53 ± 0.68. Compared with the Marfan group, controls showed higher BMI values and predominantly positive BMI-for-age z-scores, indicating a generally higher body mass relative to age- and sex-specific WHO reference standards. This distribution is consistent with the variability expected in a healthy pediatric population and includes both normal-weight and overweight individuals [7,12].

### 3.3. WHO 2007 BMI-for-Age Categories

According to the WHO 2007 BMI-for-age reference system, the two groups exhibited distinct anthropometric profiles (Table 1 and Table 3). Children with Marfan syndrome presented significantly lower BMI-for-age z-scores than controls, reflecting a leaner body habitus. However, BMI-for-age values in the Marfan group were generally within the normal range, and no consistent pattern of severe thinness was observed. In contrast, the control group showed predominantly positive z-scores, with several children presenting values above +2 SD, consistent with obesity according to WHO criteria. These findings highlight the different growth patterns of children with Marfan syndrome compared with healthy peers, with the former exhibiting a significantly leaner anthropometric profile despite maintaining BMI values that were, in most cases, within the normal range.

### 3.4. Maxillary Transverse Measurements

No systematic error was identified between repeated digital measurements, as demonstrated by the paired *t*-test analysis. The reproducibility of the measurements was supported by the use of clearly defined anatomical reference points and by the experience and training of the examiner. The magnitude of random error was calculated according to Dahlberg’s formula and resulted in a mean error of 0.42 mm, indicating an acceptable level of measurement variability.

### 3.5. Marfan Group (MG)

The mean ICW value was lower in the Marfan syndrome group than in the control group, whereas mean IMW values were similar between groups. The mean IMW/ICW ratio was also evaluated to describe the relative transverse proportions of the dental arch (Table 4). IMW values ranged from 27.6 to 34.8 mm, while ICW values ranged from 23.0 to 25.8 mm. The IMW/ICW ratio was systematically elevated (1.14–1.43), indicating disproportionate transverse development across the entire maxillary arch.

Previous studies have described craniofacial and palatal characteristics in individuals with Marfan syndrome [1,4,13,14]. In the present study, however, the observed difference was limited to the dental intercanine width, while intermolar width did not differ significantly between groups. As no skeletal measurements were performed, these findings should be interpreted as differences in transverse dental arch dimensions and cannot be used to infer underlying skeletal morphology.

### 3.6. Control Group (CG)

The CG exhibited broader and more variable transverse dimensions (Table 5). IMW values ranged from 27.3 to 34.8 mm, and ICW values from 23.7 to 31.6 mm. The IMW/ICW ratio was generally lower (1.10–1.39), indicating more balanced transverse development.

Transverse discrepancies in the CG were localized either anteriorly or posteriorly, consistent with non-syndromic patterns of dental arch variation and growth [15]. The variability observed in the CG reflects physiological differences in dental arch morphology during mixed dentition.

Descriptive statistics of anthropometric and transverse dental arch measurements are reported in Table 6. The MFS group showed a lower mean BMI-for-age z-score compared with the control group (−0.83 ± 0.87 vs. 1.53 ± 0.68). Regarding transverse dental arch measurements, children with MFS exhibited a lower mean ICW value (24.07 ± 0.72 mm) compared with controls (27.30 ± 2.33 mm), whereas mean IMW values were similar between groups (31.77 ± 2.31 mm vs. 32.80 ± 2.14 mm).

Exploratory comparisons between groups (Table 7) showed significantly lower BMI-for-age z-scores in children with MFS compared with the non-Marfan group (mean difference: 2.36; 95% CI: 1.82–2.90; *p* < 0.001). Raw BMI values were also significantly lower in the MFS group (mean difference: 4.98 kg/m^2^; 95% CI: 3.52–6.43; *p* < 0.001). Regarding transverse dental arch measurements, ICW was significantly lower in the MFS group (mean difference: 3.23 mm; 95% CI: 2.23–4.24; *p* < 0.001), whereas no significant difference was observed for IMW (mean difference: 1.03 mm; 95% CI: −0.42–2.47; *p* = 0.159). The IMW/ICW ratio was also evaluated at group level. Given the limited sample size, these findings were interpreted as exploratory.

Exploratory Pearson correlation analyses showed no significant associations between BMI-for-age z-score and transverse dental arch measurements in either group. In the control group (CG), a significant positive correlation was observed between age and BMI-for-age z-score (*r* = 0.432, *p* = 0.027), as well as between IMW and ICW (*r* = 0.598, *p* = 0.001). In the Marfan group (MG), age was strongly and positively correlated with BMI-for-age z-score (*r* = 0.844, *p* < 0.001), whereas no significant correlations were observed between age and transverse dental arch measurements or between BMI-for-age z-score and IMW or ICW. The correlation between IMW and ICW was not statistically significant in the MG (*r* = 0.327, *p* = 0.234) (Table 8).

#### 3.6.1. Dental Development and Eruption Timing

Parental reports and available clinical records indicated a history of premature exfoliation of maxillary deciduous incisors in some participants. In the MG, this was reported in 13/15 subjects (86.66%), compared with 3/26 subjects (11.53%) in the CG. These observations were based on parental history and available records and were not supported by standardized dental diagnostic assessment.

#### 3.6.2. Pubertal Development

Parental reports and available clinical records also indicated early pubertal onset in a subset of participants. In the MG, early pubertal onset was reported in 1/7 males (14.28%) and 4/8 females (50%), compared with 0/12 males and 1/14 females (6.25%) in the CG. These observations were not clinically verified and are therefore reported descriptively without formal comparison between groups.

## 4. Discussion

The present study provides a descriptive comparison of BMI-for-age and transverse dental arch dimensions in children with Marfan syndrome (MFS) and a non-Marfan pediatric comparison group. Given the retrospective design and the relatively small MFS cohort, the findings should be considered preliminary and interpreted within the limitations of the study.

From an anthropometric perspective, children with MFS showed significantly lower BMI-for-age z-scores than the comparison group. Most children with MFS nevertheless remained within the expected range for age and sex, suggesting a generally leaner anthropometric profile rather than a uniform pattern of undernutrition. This finding is consistent with the tall and slender body habitus commonly described in MFS and further supports the importance of considering age- and sex-adjusted measures when evaluating growth in affected children [9,10,11].

Regarding transverse dental arch dimensions, the MFS group showed a significantly smaller mean intercanine width (ICW), whereas the difference in intermolar width (IMW) did not reach statistical significance. These findings suggest that the observed difference was more evident in the anterior transverse dental dimension than in the posterior dimension. The IMW/ICW ratio was also higher in the MFS group and was considered a descriptive measure of dental arch proportions. However, given the overlap between groups and the exploratory nature of the analysis, this ratio should not be interpreted as evidence of generalized transverse deficiency.

Previous studies have described craniofacial and palatal characteristics in individuals with MFS [4,13,15,16]. However, the present study was based exclusively on dental measurements obtained from digital models and did not include skeletal or cephalometric assessment. Therefore, the observed differences in ICW and IMW cannot be used to establish an underlying skeletal constriction, palatal-vault deficiency, or a diagnosis of maxillary transverse deficiency. The present findings should consequently be interpreted specifically in terms of transverse dental arch dimensions.

The exploratory correlation analysis did not demonstrate significant associations between BMI-for-age z-score and either IMW or ICW in the MFS or control group. These results do not exclude the possibility of an association, particularly given the small sample size and the resulting imprecision of the correlation estimates. Rather, they indicate that no clear linear relationship could be identified in this cohort. The significant correlation observed between age and BMI-for-age z-score in the MFS group further highlights the importance of considering developmental age when interpreting anthropometric characteristics in pediatric patients.

Retrospective observations concerning dental exfoliation and pubertal timing were also recorded. Previous studies have described associations between dental eruption patterns and dental arch development [17,18]. However, in the present study, these observations were based on parental reports and available clinical records and were not supported by standardized dental or endocrinological assessments.

They were therefore considered exploratory observations and should not be interpreted as evidence of differences in dental developmental timing or pubertal development between groups.

Overall, the present findings indicate that children with MFS in this cohort had a leaner BMI-for-age profile and smaller mean ICW compared with the non-Marfan comparison group, while IMW did not differ significantly. These observations provide preliminary quantitative information on transverse dental arch dimensions in pediatric MFS and may serve as a basis for future research. Larger prospective and multicenter studies incorporating standardized longitudinal growth assessment and comprehensive craniofacial measurements, including skeletal parameters, are warranted to further investigate these relationships.

## 5. Study Limitations

Some limitations should be considered when interpreting the present findings. The relatively small Marfan syndrome cohort, reflecting the rarity of the condition, limits statistical power and supports the exploratory nature of the analyses. The retrospective design and clinic-based control group may introduce variability and residual confounding related to age, sex, developmental stage, and previously recorded anthropometric data. BMI-for-age z-scores were used to account for age- and sex-related differences in pediatric growth. Correlation analyses should be interpreted cautiously because of the small sample size and the resulting imprecision of the estimates. Secondary observations regarding dental eruption and pubertal timing were based on parental reports and available records and may therefore be affected by recall or misclassification bias. Finally, transverse assessment was based exclusively on dental measurements at a single time point and cannot establish underlying skeletal morphology or a diagnosis of transverse maxillary deficiency. Further prospective studies with larger cohorts and standardized craniofacial assessments are warranted to confirm these preliminary findings.

## 6. Conclusions

Growing children with Marfan syndrome showed a leaner anthropometric profile, characterized by lower BMI-for-age values than the non-Marfan comparison group, together with a significantly smaller mean intercanine width. The difference in intermolar width was not statistically significant. Exploratory correlation analyses did not demonstrate significant associations between BMI-for-age z-score and transverse dental arch measurements in either group. These findings should be interpreted cautiously because of the small sample size and retrospective study design. Further longitudinal and multicenter studies with larger cohorts are warranted to better characterize the relationship between growth patterns and craniofacial development in children with Marfan syndrome.

## Figures and Tables

**Table 1:** Anthropometric characteristics of the MG, WHO 2007 BMI-for-Age Assessment (z-score).

Age (y,m)	Gender	Height (cm)	Weight (kg)	BMI (kg/m^2^)	Z-Score
6,1	F	137.5	25.0	13.22	−1.53
6,5	M	139.5	26.0	13.36	−1.72
7,3	F	141.0	27.0	13.59	−1.3
7,2	M	143.0	28.0	13.69	−1.49
8,3	F	145.0	29.0	13.79	−1.3
8,7	F	147.0	30.0	13.88	−1.31
8,9	M	149.0	31.0	13.96	−1.52
9	F	150.0	32.0	14.22	−1.34
9,2	M	152.0	33.0	14.25	−1.01
9,5	F	154.0	34.0	14.35	−1.19
9,7	M	142.5	31.5	15.52	−0.49
10,3	M	144.0	34.0	16.40	−0.11
10,4	F	145.5	37.0	17.48	0.3
10,8	F	147.5	40.0	18.38	0.57
11,2	M	149.0	43.0	19.37	1.02

**Table 2:** Comparison of BMI and BMI-for-age z-scores between children with MG and CG.

	MG (n = 15)	CG (n = 26)
BMI (kg/m^2^)	15.03 ± 1.96	20.01 ± 2.36
BMI-for-age z-score	−0.83 ± 0.87	1.53 ± 0.68

**Table 3:** Anthropometric characteristics of the CG, WHO 2007 BMI-for-Age Assessment (z-score).

Age (y,m)	Gender	Height (cm)	Weight (kg)	BMI (kg/m^2^)	Z-Score
6,3	F	124.0	23.0	14.97	−0.22
6,4	M	126.5	25.0	15.62	0.19
6,8	M	128.0	26.5	16.17	0.51
7	M	129.5	27.5	16.40	0.6
7,1	F	131.0	32.0	18.66	1.56
7,6	M	132.5	34.0	19.38	2.01
7,6	F	134.0	36.0	20.06	1.96
7,9	M	136.0	33.0	17.83	1.25
8,3	F	133.0	37.0	20.92	2.03
8,5	M	140.5	40.0	20.27	2.13
8,5	F	123.5	32.0	20.98	2
8,7	F	125.0	30.0	19.20	1.57
8,8	F	126.5	31.0	19.36	1.45
8,9	F	128.0	32.0	19.53	1.47
9,1	M	129.0	36.0	21.62	2.33
9,2	F	130.5	37.0	21.70	2.05
9,3	F	131.5	38.0	21.99	2.09
9,4	F	133.0	39.0	22.06	2.04
10,1	F	134.0	40.0	22.28	2.04
10,2	F	135.0	41.0	22.48	2.22
10,4	M	136.0	33.0	17.83	0.62
10,6	M	130.0	36.0	21.32	1.82
10,9	F	127.5	38.0	23.39	1.14
11,3	M	132.0	40.0	22.94	2.05
11,6	M	139.0	42.0	21.72	1.67
11,7	F	141.5	43.0	21.50	1.3

**Table 4:** Linear measurements and ratios of MG.

Age (y,m)	Gender	IMW (mm)	ICW (mm)	Ratio
6,1	F	28.7	23.9	1.20
6,5	M	32.6	22.9	1.42
7,3	F	33.2	24.1	1.37
7,2	M	34.5	24.3	1.41
8,3	F	29.5	24.7	1.19
8,7	F	27.6	24.1	1.14
8,9	M	34.8	25.8	1.34
9	F	29.3	23	1.27
9,2	M	34.2	23.9	1.43
9,5	F	34.3	24.2	1.41
9,7	M	32.9	24.9	1.32
10,3	M	31.1	23.5	1.32
10,4	F	30.1	24.1	1.24
10,8	F	32.3	23.7	1.36
11,2	M	31.5	23.9	1.31
**Mean**		31.8	24.1	1.31
**SD**		2.3	0.7	0.09

**Table 5:** Linear measurements and ratios of CG.

Age (y,m)	Gender	IMW (mm)	ICW (mm)	Ratio
6,3	F	34.4	30.5	1.12
6,4	M	28	24.9	1.12
6,8	M	33.5	29.3	1.14
7	M	31.9	28.6	1.11
7,1	F	34.4	28.8	1.19
7,6	M	34.8	31.6	1.10
7,6	F	34.3	29.8	1.15
7,9	M	33	27.2	1.21
8,3	F	31.9	23.8	1.34
8,5	M	34.5	31.2	1.1
8,5	F	27.3	24.3	1.12
8,7	F	28.6	23.7	1.20
8,8	F	32.9	25.7	1.28
8,9	F	34.7	24.9	1.39
9,1	M	33.9	25.7	1.31
9,2	F	30.5	26.9	1.13
9,3	F	31.6	25.6	1.23
9,4	F	34.4	27.7	1.24
10,1	F	34.5	29.5	1.16
10,2	F	31.7	26.4	1.20
10,4	M	34.2	25.7	1.33
10,6	M	33	29.2	1.13
10,9	F	32.1	26	1.23
11,3	M	34.3	30.1	1.13
11,6	M	34.8	25.8	1.34
11,7	F	33.6	26.9	1.24
**Mean**		32.8	27.3	1.19
**SD**		2.1	2.3	0.08

**Table 6:** Descriptive summary of anthropometric and transverse dental arch measurements in the MF and CG.

Variable	Group	N	Mean	SD	Std. Error Mean
**BMI-for-age z-score**	CG	26	1.530	0.680	0.133
	MG	15	−0.830	0.870	0.225
**IMW (mm)**	CG	26	32.800	2.144	0.421
	MG	15	31.773	2.309	0.596
**ICW (mm)**	CG	26	27.300	2.332	0.457
	MG	15	24.067	0.718	0.185

**Table 7:** Exploratory comparison of anthropometric and transverse dental arch measurements between CG and MG.

Variable	Mean Difference (CG–MG)	95% CI	t	df	*p*-Value
BMI-for-age z-score	2.362	1.822–2.902	9.029	24.091	<0.001
BMI (kg/m^2^)	4.976	3.518–6.435	6.902	39	<0.001
IMW (mm)	1.027	−0.419–2.472	1.436	39	0.159
ICW (mm)	3.233	2.229–4.238	6.552	32.328	<0.001

**Table 8:** Pearson correlations between age, BMI-for-age z-score, intermolar width (IMW), and intercanine width (ICW) in the control and Marfan syndrome groups.

Group	Variable	Age	BMI-for-Age z-Score	IMW	ICW
**Control group (CG)**	**Age**	1	0.432 * (0.027)	0.189 (0.355)	−0.189 (0.356)
	**BMI-for-age z-score**	0.432 * (0.027)	1	0.079 (0.700)	−0.025 (0.904)
	**IMW**	0.189 (0.355)	0.079 (0.700)	1	0.598 * (0.001)
	**ICW**	−0.189 (0.356)	−0.025 (0.904)	0.598 * (0.001)	1
**Marfan group (MG)**	**Age**	1	0.844 * (<0.001)	0.034 (0.903)	0.077 (0.786)
	**BMI-for-age z-score**	0.844 * (<0.001)	1	−0.068 (0.809)	−0.104 (0.713)
	**IMW**	0.034 (0.903)	−0.068 (0.809)	1	0.327 (0.234)
	**ICW**	0.077 (0.786)	−0.104 (0.713)	0.327 (0.234)	1

**Note:** Values are Pearson correlation coefficients (*r*), with two-tailed *p*-values in parentheses. *p* < 0.05 is considered statistically significant. *p* < 0.01 is considered highly significant. Abbreviations: BMI, body mass index; IMW, intermolar width; ICW, intercanine width; CG, control group; and MG, Marfan group. * significant.

## Data Availability

The data presented in this study are available upon request from the corresponding author. The data are not publicly available due to privacy and ethical reasons.
